# Effects of visual landscape on subjective environmental evaluations in the open spaces of a severe cold city

**DOI:** 10.3389/fpsyg.2022.954402

**Published:** 2022-09-29

**Authors:** Jianru Chen, Yumeng Jin, Hong Jin

**Affiliations:** ^1^Hong Kong Huayi Design Consultants (S.Z) LTD., Shenzhen, China; ^2^School of Architecture, Harbin Institute of Technology, Harbin, China; ^3^School of Architecture and Urban Planning, Suzhou University of Science and Technology, Suzhou, China

**Keywords:** severe cold city, urban open space, visual landscape, subjective environmental evaluation, visual landscape evaluation

## Abstract

The environmental quality and subjective environmental evaluations in urban open spaces are essential. In this study, the effects of building, green, and water landscapes, which are typical visual landscapes, on the subjective environmental evaluations (including thermal sensation and comfort, and overall comfort) in different seasons were analyzed by conducting questionnaire surveys and field measurements in a severely cold city. It was found that the visual landscapes significantly affected subjective environmental evaluations in winter and summer, but there were no effects in the transitional season. In summer, compared with the building and green landscape, the thermal sensation vote in the water landscape was the lowest at 0.4, and the differences were 0.3∼1.0. However, the thermal comfort vote in the water landscape was found to be 0.6 times higher. In winter, the thermal sensation and comfort votes in the water landscape were the lowest, the average evaluation under different UTCI was –2.2, and the results were similar for the overall comfort evaluation. In addition, the subjects believed that green and water landscapes improved thermal comfort and had more significant effects on improving the environmental temperature in the three seasons. Additionally, visual landscape evaluations significantly affect subjective environmental evaluations in summer than in the winter and transitional season; the higher the visual landscape evaluation, the better the thermal and overall comfort.

## Introduction

The environmental quality and the user’s subjective environmental evaluations in urban open spaces are essential for judging their quality. As the thermal environment always significantly affects human perception, thermal comfort is critical in subjective environmental evaluations. According to the thermal comfort theory, six main factors affect people’s thermal comfort, including, air temperature, average radiation temperature, airflow velocity, relative humidity, clothing thermal resistance, and physiological metabolic rate. The ASHRAE standard states that thermal comfort is a psychological state that expresses satisfaction with the thermal environment, which is evaluated through subjective evaluation (ASHRAE, 2017). It is not only affected by the physical conditions of the environment, but also by other sensory stimulants, individual preferences, and psychological factors. Therefore, people’s perception of the thermal environment is based on the interactions between the body’s multiple sense organs. Previous studies on subjective environmental evaluations, particularly thermal comfort, mainly focused on the effects of the physical environment on comfort evaluation and aimed to improve people’s subjective comfort by improving the objective environment (Dimoudi and Nikolopoulou, 2003; Huang et al., 2008; Bowler et al., 2010; Zheng et al., 2016; Tong et al., 2017; Tan et al., 2020; Zheng et al., 2018; Nishimura et al., 1998; Saaroni and Ziv, 2003; Syafii et al., 2017). When people receive environmental information, over 85% is obtained by vision, which is the main sense that humans use to process the surrounding environment (Weissmann, 2016). Vision directly affects people’s psychological regulations, thereby affecting their subjective environmental evaluations. Therefore, the effects of visual factors on outdoor human subjective environmental evaluations should be studied.

Some studies regarding the effects of visual factors on subjective environmental evaluations were conducted from the perspective of the interior space environment and color. Ko et al. discussed the effect of window scenery on people’s indoor thermal comfort, and the results showed that, in a warm environment, subjects with a visual connection to the outdoors through windows felt cooler, more comfortable, and happier than subjects without windows to look out Ko et al. (2020). Itten and Clark reported that subjects felt colder in blue and blue-green rooms (Clark, 1975; Itten, 1987). Greene and Pedersen discussed the effect of spatial color tone on people’s temperature perception, and found that the subjects’ thermal comfort differed in red, blue, and white rooms, and the estimation of temperature differed when the ambient temperature was the same (Pedersen et al., 1978; Greene and Bell, 1980). Wang et al. reported that, at different temperatures, color affects thermal sensation in indoor environments. Warm colors make people feel warmer, and cool colors make people feel cooler than neutral colors (Wang et al., 2018). Brambilla et al. reported that cool light can control people’s thermal sensation and expand their acceptable temperature range (Brambilla et al., 2020).

The effect of visual properties of the outdoor environment on subjective comfort is still largely unknown. Knez et al. regard physical attributes, such as form (structure and openness), material (surface properties), naturalness (degree of artificiality), and location (spatial dimension), as indirect psychological factors affecting people’s thermal comfort (Knez et al., 2009). Lenzholzer et al. demonstrated that people’s perception of the spatial environment would affect their thermal comfort evaluation (Lenzholzer and van der Wulp, 2010), while Klemm et al. suggested that urban green space design positively affects people’s thermal comfort perception. Rich vegetation types and height variation in the visual field can improve people’s thermal comfort experience. Klemm et al. also studied the long-term thermal comfort perception of residents during warm summers, and reported that people subjectively thought that green urban environments were more comfortable than water or building environments (Klemm et al., 2015a,b). Schnell et al. demonstrated that tree-lined urban boulevards positively affected people’s thermal comfort (Schnell et al., 2016), while Mazzota compared the effects of new permeable paving materials, grass, and mixed grass and concrete paving on people’s thermal comfort, and found that the subjects were more satisfied with the thermal comfort of mixed paving and grass than the new permeable paving materials (Mazzotta and Mutani, 2015). Rosso et al. (2015) demonstrated that grass is superior to gravel and asphalt in terms of thermal comfort and visual comfort.

In the existing research on the relationship between visional perception and environmental perception, most researchers were inclined to explore the effects of visual factors in the indoor environment, such as indoor spacial elements, form, lighting, color, etc. In terms of the outdoor environment, most researchers were inclined to explore the effects of single factors, such as surface materials, paving types, and site morphology, or the effects of visual perceptions on subject environmental perceptions in a single season, focusing on the effects on human thermal comfort. However, the influence mechanism of visual landscapes on overall comfort and the environmental perception across multi-season was still unclear.

With regard to overall comfort, it is related to multiple factors, such as thermal environment, acoustic environment, light environment, air, scenery, smell, etc. (Yang and Kang, 2005; Huang et al., 2012; Guan et al., 2020). In order to explore whether visual factors occupy an important position among the many factors that affect overall comfort, a preliminary experiment was conducted, which polled the factors affecting the overall comfort of the subjects. The results showed that visual factors accounted for 12.3% of the effects on the outdoor people’s overall comfort, next only to the thermal environment, light environment, and air quality (16.3, 16.3, and 13.1%, respectively). Meanwhile, there was a significant positive correlation between overall comfort and thermal comfort (*P* < 0.01). This shows that people in outdoor activities generally think that visual factors are critical. Visual factors have a certain effect on the overall comfort evaluation, and thermal comfort also significantly affects the overall comfort evaluation. Therefore, it is necessary to explore the relationship between visual landscape and overall comfort.

Considering the limitations of previous studies and the necessity of improving outdoor human comfort from a visual perspective, urban open spaces with different visual landscapes were selected to conduct the questionnaire surveys of subjective environmental evaluations and to monitor the field environment in winter, transitional seasons, and summer. This study aimed to analyze the effects of the urban visual landscape on subjective environmental evaluations (including thermal sensation, thermal comfort, and overall comfort evaluation) and to explore the variations in the effects of the same visual landscape on subjective environmental evaluations due to the changes in different seasons. The collected data objectively reflected the subjective environmental evaluations of urban open spaces in different seasons to provide a new basis for urban open space design. It also promoted focusing on the relationship between visional perception and environmental perception.

## Materials and methods

### Locations

This study was conducted in Harbin, a typical city in a severe cold region in China. According to the Koppen climate classification, Harbin belongs to the “Hot Summer Continental (Dwa),” where the average temperature in the coldest month is below 0°C and above –38°C, and the average temperature in the hottest month is 22°C and above.

**FIGURE 1:**
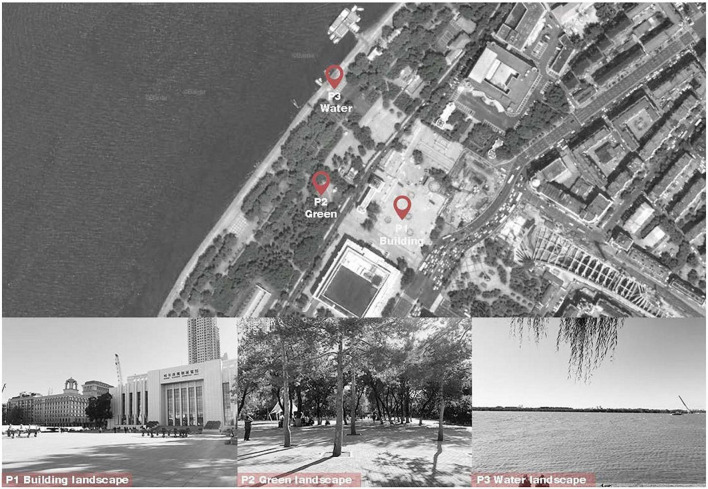
Locations of questionnaire survey and the measurement sites. The figure of Locations adapted from Baidu Maps, available at https://map.baidu.com/.

Typical visual landscapes were summarized and selected to study the effects of visual landscapes in urban open spaces on subjective environmental evaluations. Visual landscape refers to all kinds of landscapes viewed by the viewers, and typical visual landscapes refer to the landscape most seen in urban open spaces, which are the environmental art spaces formed by buildings, gardens, water, squares, roads, and other elements. This study selected architecture, green, and water landscapes as the typical landscapes in urban open spaces. As shown in Figure 1, three representative urban landscapes of these types located in the central area of the city were selected as measurement points, including People’s Square (P1), Green Leisure Square in Jiuzhan Park (P2), and Riverside Landscape Trail Square (P3). The landscape of P1 mainly included buildings and urban roads, with almost no natural landscape, and was considered as a building landscape. P2 was located inside a green landscape, while P3 was situated in the water landscape trail along the river. The three measurement points were relatively close to one other; thus, the climatic conditions were relatively consistent.

By calculating the viewing area of the panoramic images of the three squares (except the sky area), it can be seen that the building landscape area of P1 is 48∼60%, the green landscape area of P2 is 51∼64%, and the water landscape area of P3 is 40∼53%.

### Questionnaires

The questionnaire survey was used to conduct a subjective environmental evaluation in urban open spaces, and the questionnaire was divided into three sections: basic information, environmental evaluation, and visual landscape evaluation. The basic information included the subjects’ gender, age, and clothing; the environmental evaluation included the thermal sensation vote (TSV), thermal comfort vote (TCV), and overall comfort vote (OCV) in different visual landscapes; and the visual landscape evaluation mainly included subjects’ evaluation of the aesthetics, likability, satisfaction, and subjective effect of improving temperature.

Based on relevant studies, especially for environmental evaluations in severe cold regions, a 7-point Likert scale was used for TCV and OCV. Considering the extreme heat and cold conditions around the year, “very hot” and “very cold” were added, so a 9-point Likert scale was used for TSV, which could assess the subjects’ thermal sensation levels more accurately (Jin et al., 2019, 2020). As there were no extreme visible changes in the landscapes and the over-detailed evaluation scale in the visual landscape evaluation would confuse the subjects and affect their subjective evaluation, a 5-point Likert scale was used for visual landscape evaluation.

### Measurements

During the field measurement process, BES-01 temperature recorders were used to measure the blackball temperature. The blackball diameter was 0.08 m, and the scattering coefficient of the surface material was 0.95. BES-02 temperature and humidity recorders were used to measure the air temperature and humidity, and portable Kestrel 5500 weather stations were used to record the wind velocity and orientation. The characteristics of the instruments are shown in Table 1.

**TABLE 1:** Characteristics of the measurement instruments.

Type	Range	Precision	Sampling period
BES-01 temperature recorder	–30 to 50°C	± 0.5°C	10 s–24 h
BES-02 temperature and humidity recorder	–30 to 50°C 0–99% RH	± 0.5°C ±3% RH	10 s–24 h
Kestrel 5500 weather station	0.4–40 m/s 0–360°	±0.1 m/s ±5°	2 s–12 h

**FIGURE 2:**
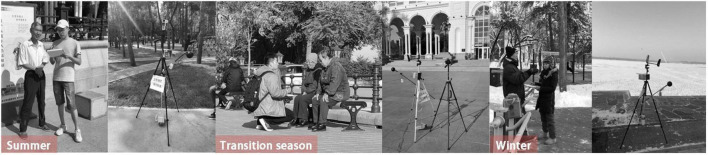
Questionnaire survey sites and measurement instrument layout in each season.

All of the instruments were calibrated before field measurement. The temperature recorders were placed inside a radiation-resistant aluminum hood to prevent interference from solar radiation and winds. Figure 2 shows the field measurement instruments and equipment. The measurement instruments were set up in accordance with ISO 7726 and held by the tripods at the height of 1.2 m above the ground (International Standard Organization [ISO], 1998), which complies with the measurement standards. The data sampling interval was 1 min.

### Procedures and subjects

July, October, and December were selected to represent the summer (hot), autumn (transitional), and winter (cold) seasons. Five days were selected for questionnaire survey and field measurement under the typical meteorological conditions in each season. The subjective questionnaire surveys and thermal environment monitoring were conducted between 9:00 and 17:00, during which outdoor activities were most frequent. The questionnaires were conducted simultaneously at the three measurement points. The questionnaire surveys were conducted near the measurement points to ensure accurate monitoring of the objective environment conditions.

All the subjects were recruited on-site, informed about the purpose of the questionnaire, and volunteered to participate in the survey. They claimed to be in good health and eyesight, sitting or standing instead of doing strenuous exercise before filling out the questionnaire. Each subject completed only one questionnaire in a single landscape scene, and there were no subjects who participated in the questionnaire repeatedly in different seasons. It took about 3∼4 min to complete a questionnaire.

A total of 653 valid questionnaires were collected for this study: 183 in winter, 193 in the transitional season, and 277 in summer. All the subjects were within different landscapes, informed about the purpose of the questionnaire, and volunteered to participate in the survey. The statistical power level and effect size of each season were calculated as follows: summer (1 – β = 0.96, α = 0.05, effect size = 0.25), transitional season (1 – β = 0.87, α = 0.05, effect size = 0.25), and winter (1 – β = 0.86, α = 0.05, effect size = 0.25); the sample size in each season passed the effect size test. The subjects included 58% men and 42% women, their average age and metabolic rate were 53 years and 1.4 met, respectively, and their average clothing insulation levels were 1.77, 0.78, and 0.40 Clo in winter, the transitional season, and summer, respectively.

### Thermal comfort indices

As the outdoor thermal environment was unstable, there were differences in the thermal environment, clothing, exercise status, and other aspects when the subjects were surveyed. Therefore, the universal thermal climate index (UTCI) was selected to assess the physiological effects of the thermal environment, which is expressed as an equivalent ambient temperature (°C) of a reference environment that provides the same physiological response of a reference person as the actual environment (Jendritzky et al., 2012; Pantavou et al., 2013; Jin et al., 2019). As humans have different thermal experiences and expectations in different regions, their thermal environment evaluations vary. Therefore, the UTCI range must be corrected at different thermal stress levels. The revised UTCI thermal stress range of Harbin is adopted in this study (Jin et al., 2019), as shown in Table 2.

**TABLE 2:** The revised Harbin universal thermal climate index (UTCI) thermal stress range.

Stress category	UTCI/°C
Extreme cold stress	Below –30.2
Very strong cold stress	–30.2∼–25.6
Strong cold stress	–25.6∼–18.3
Moderate cold stress	–18.3∼–7.2
Slight cold stress	–7.2 ∼ –3.8
No thermal stress	–3.8∼+23.0
Moderate heat stress	+23.0∼+29.1
Strong heat stress	+29.1∼+40.9
Very strong heat stress	+40.9∼+49.4
Extreme heat stress	Above +49.4

The collected samples were grouped according to different thermal stress levels based on the revised UTCI range corresponding to different thermal stresses. Subjects were grouped according to thermal stress, and these thermal stress ranges corresponded to different categories, as shown in Table 3.

**TABLE 3:** Sample classification according to the universal thermal climate index (UTCI) thermal stress interval.

Stress category	Winter		Transitional season		Summer	
	Range/°C	Average value/°C	Range/°C	Average value/°C	Range/°C	Average value/°C
Strong cold stress	–23.1∼–18.4	–19.8				
Moderate cold stress	–18.2∼–7.4	–14.4				
Slight cold stress	–7.1∼–4.0	–5.8				
No thermal stress	–3.7∼–2.1	–3.0	6.0∼22.7	15.4		
Moderate heat stress			23.1 ∼25.1	23.7	27.3∼28.6	27.9
Strong heat stress					29.2∼39.8	34.3
Extreme heat stress					40.2∼44.1	42.0

Owing to the influence of the on-site thermal environment, different landscapes, and randomness of the samples during field investigation, the UTCI distribution of the collected samples was uneven, and there was no guarantee that UTCI data could be collected for all thermal stress levels. Therefore, only the thermal stress categories with a uniform distribution of samples in each season were extracted in this study. In winter, strong (–19.8°C) and moderate (–14.4°C) cold stress was observed, no thermal stress (15.4°C) was observed in the transitional season, and strong heat stress (34.3°C) was observed in the summer. All data were the average values obtained at different thermal stress levels.

## Results

### Effects of visual landscape on thermal sensation vote

Table 4 shows the significance analysis of the effects of UTCI and landscape scenes on subjective environmental evaluations. The analysis of variance (ANOVA) for TSV indicated that the effect of UTCI on TSV was significant in all three seasons (*P* < 0.01). The visual landscape significantly affected TSV in summer and winter (*P* < 0.01), but did not affect TSV in the transitional season (*P* > 0.05). The interaction between UTCI and visual landscape only affected TSV in summer (*P* < 0.05). The *post hoc* test of the ANOVA for visual landscape and TSV (Table 5) indicated that there was no difference between the TSV in the building and green landscapes in winter and summer (*P* > 0.05), but both of their TSV values were significantly different from those of the water landscape (*P* < 0.01).

**TABLE 4:** Significance analysis of the effects of universal thermal climate index (UTCI) and landscape scenes on subjective environmental evaluations.

Subjective environm-ental evaluation	Winter			Transitional season			Summer		
	UTCI	Landscape	UTCI* Landscape	UTCI	Landscape	UTCI* Landscape	UTCI	Landscape	UTCI* Landscape
TSV	0.002	**0.002**	0.623	**0.004**	0.728	0.493	**0.000**	**0.000**	**0.015**
TCV	**0.011**	**0.008**	0.500	**0.038**	0.645	0.980	**0.000**	**0.033**	0.122
OCV	**0.020**	**0.044**	0.162	**0.035**	0.320	0.743	**0.000**	**0.000**	0.733

Bold font indicates significant analysis results.

**TABLE 5:** *Post hoc* test of the difference in the thermal sensation vote (TSV) values between landscapes.

Subjective environmental evaluation	*Post hoc* test	Winter		Summer	
		Mean difference	Significance	Mean difference	Significance
TSV	Building vs Green	0.0	0.920	–0.4	0.054
	Building vs Water	1.1	**0.000**	0.7	**0.000**
	Green vs Water	1.2	**0.000**	1.1	**0.000**

Bold font indicates significant analysis results.

**FIGURE 3:**
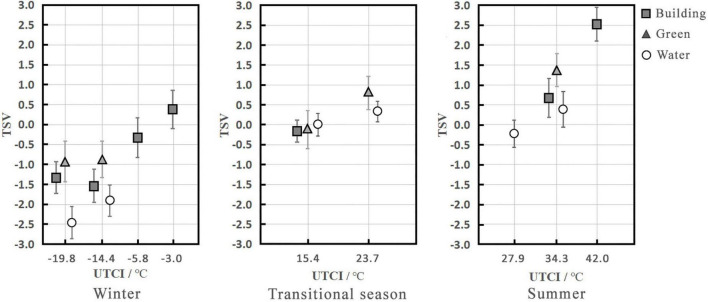
Mean TSV under different thermal stress levels and visual landscapes (abscissa is the categorical variable).

To explore the effects of the visual landscape on subjective evaluations, such as thermal sensation and comfort, it was necessary to ensure that the subjects’ thermal environment conditions were consistent. Figure 3 shows the TSV values under different UTCI levels and visual landscapes. Strong and moderate cold stress were the main thermal stress levels experienced by subjects in winter, with average UTCI values of –19.8 and –14.4°C, respectively. The TSV values for the different landscapes increased in the following order: water landscape (TSV = –2.5 and –1.9) < building landscape (TSV = –1.3 and –1.5) < green landscape (TSV = –0.9). The TSV values in the water landscape tended to be cold and cool, while those in the building and green landscapes tended to be slightly cold; the TSV in the water landscape was the lowest. In the transitional season, the subjects mainly experienced no thermal stress, and the average UTCI was 15.4°C. The visual landscape did not significantly affect the TSV, which tended to be neutral (building, TSV = –0.2; green, TSV = –0.1; water, TSV = 0.0). In summer, the subjects experienced strong heat stress, and the average UTCI value was 34.3°C. The TSV performance of the different landscapes increased in the following order: water landscape (TSV = 0.4) < building landscape (TSV = 0.7) < green landscape (TSV = 1.4). The TSV of the water and building landscapes tended to be neutral, while that of the green landscape tended to be slightly warm. Although TSV in the water landscape was still the lowest, it increased the subjects’ comfort in summer.

Under the same thermal stress level, the TSV of the water landscape was lower than those of the building and green landscapes in winter and summer, with maximum differences of 1.5 and 1.0, respectively; however, there were no differences between the different visual landscapes in the transitional season, and the subjective environmental evaluations varied in different seasons. In winter, a low TSV in the water landscape was associated with lower comfort. However, a low TSV in the water landscape was associated with increased comfort in summer. The results of previous studies reported that water in cities can significantly regulate the surrounding microclimate, and urban water bodies can improve human comfort in the surrounding areas, and this effect is clearer in spring and summer than in winter (Robitu et al., 2006; Xu et al., 2010; Theeuwes et al., 2013; Syafii et al., 2017). Therefore, water bodies exert cooling and humidification effects, and improve the surrounding microclimate. When people move around the water body in winter, although they experience the same thermal stress level as that in the other visual landscapes, they still perceive a cooling effect from the water body, leading them to subjectively believe that the temperature of the water landscape is the lowest.

### Effects of visual landscape on thermal comfort vote

The results of the ANOVA test for TCV in Table 4 show that UTCI affected TCV in all three seasons (*P* < 0.05). The visual landscape significantly affected TCV in winter and summer (*P* < 0.01 and < 0.05, respectively). The interaction between UTCI and visual landscape did not affect TCV in all three seasons (*P* > 0.05). The *post hoc* test for the ANOVA between the visual landscape and TCV in Table 6 found that the TCV of both the building and green landscapes were significantly different from the water landscape (*P* < 0.05) in winter and summer, but there was no significant difference between these two landscapes (*P* > 0.05).

**TABLE 6:** *Post hoc* test of the difference between thermal comfort vote (TCV) and landscapes.

Subjective environmental evaluation	*Post hoc* test	Winter		Summer	
		Mean difference	Significance	Mean difference	Significance
TCV	Building vs Green	0.2	0.678	–0.5	0.084
	Building vs Water	0.8	**0.001**	–1.2	**0.000**
	Green vs Water	0.6	**0.014**	–0.6	**0.025**

Bold font indicates significant analysis results.

**FIGURE 4:**
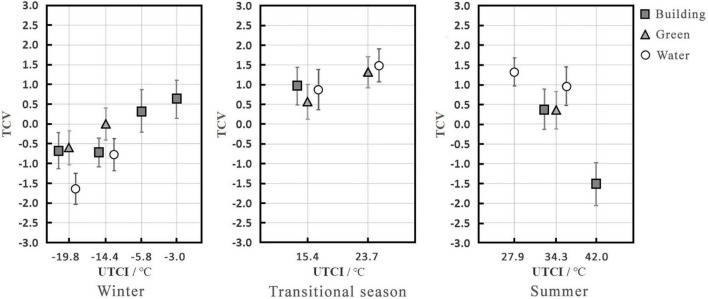
Mean TCV under different thermal stress levels and between different visual landscapes (abscissa is a categorical variable).

Figure 4 shows the TCV under different UTCI values and in visual landscapes. In winter, at UTCI values of –19.8 and –14.4°C, the TCV increased in the following order: water landscape (TCV = –1.6 and –0.8) < building landscape (TCV = –0.7) < green landscape (TCV = –0.6 and 0.0). At a UTCI value of –19.8°C, the TCV of the water landscape was lowest and tended to be uncomfortable. Although the TCV of the green landscape was slightly higher than that of the building landscape, they also both tended to be slightly uncomfortable. At a UTCI of –14.4°C, the TCV of the building and water landscapes were very similar and tended to be slightly uncomfortable, while that of the green landscape was highest and tended to be neutral. In the transitional season, there was no significant difference in the TCV of the three visual landscapes, and they all tended to be slightly comfortable. In summer, at a UTCI of 34.3°C, the TCV increased in the following order: green landscape (TCV = 0.4) < building landscape (TCV = 0.4) < water landscape (TCV = 1.0). The TCV of the building and green landscapes were similar, with both tending to be neutral, while that of the water landscape was the highest and tended to be slightly comfortable.

Regarding the effects of visual landscape on TCV, under the same thermal stress level, the TCV of the water landscape was lowest in winter and highest in summer, and there was no apparent difference between the building and green landscape. At the same time, it was found that the lower the temperature, the greater the effect tendency on TCV in the water landscape, and the lower the TCV. The correlation analysis of TSV and TCV found a significant correlation between them (*P* < 0.01), which also explains why the effect of the visual landscape on TCV was the same as that on TSV.

### Effects of visual landscape on overall comfort vote

The results of the ANOVA between UTCI and OCV presented in Table 4 indicate that UTCI affected OCV in the three seasons (*P* < 0.05). The visual landscape affected the OCV in summer and winter (*P* < 0.05), but had no effect in the transitional season (*P*>0.05). The results of the *post hoc* test for the ANOVA between visual landscape and OCV presented in Table 7 were the same as those for TSV and TCV; i.e., both the building and green landscapes were significantly different from the water landscape in winter and summer (*P* < 0.01). However, there was a significant difference between the building and green landscapes in summer (*P* < 0.05), and no difference between the two in winter (*P*>0.05).

**TABLE 7:** *Post hoc* test of the differences in the OCV between landscapes.

Subjective environmental evaluation	*Post hoc* test	Winter		Summer	
		Mean difference	Significance	Mean difference	Significance
OCV	Building vs Green	0.4	0.148	0.5	**0.004**
	Building vs Water	1.1	**0.000**	–0.9	**0.000**
	Green vs Water	0.7	**0.002**	–1.4	**0.000**

Bold font indicates significant analysis results.

**FIGURE 5:**
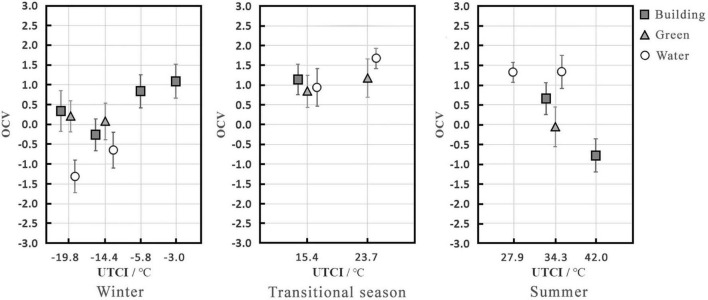
Mean OCV under different thermal stress levels and between visual landscapes (abscissa is a categorical variable).

Figure 5 shows the OCV values under different UTCI and visual landscapes. In winter, at a UTCI of -19.8°C, the OCV increased in the following order: water (OCV = –1.3) < green (OCV = 0.2) < building landscape (OCV = 0.3). The OCV of the water landscape was lowest and tended to be uncomfortable, while that of the building landscape was somewhat higher than that of the green landscape with both being between neutral and slightly comfortable. At a UTCI of –14.4°C, the OCV increased in the following order: water (OCV = –0.7) < building (OCV = –0.3) < green landscape (OCV = 0.1). The OCV in the water landscape was still lowest and tended to be uncomfortable, while that in the green landscape was slightly higher than that in the building landscape, with both tending to be neutral. In the transitional season, there was no significant difference in the OCV between the three visual landscapes (building, OCV = 1.2; green, OCV = –0.8; water, OCV = 0.9), with the maximum difference between the three of 0.2, and they all tended to be slightly comfortable. In summer, at a UTCI of 34.3°C, the OCV increased in the following order: green (OCV = –0.1) < building (OCV = 0.7) < water landscape (OCV = 1.4); the OCV in the water landscape was between slightly comfortable and comfortable, while that in the green landscape was the lowest at a neutral level, and the difference between the two reached 1.4. The OCV of the building landscape was in the middle, with a value of approximately 0.7, which tended to be slightly comfortable.

Regarding the effects of visual landscape on OCV, under the same thermal stress level, the water landscape was the most uncomfortable in winter, and there was no notable difference between the building and green landscapes, which were both more comfortable than the water landscape. The water landscape was the most comfortable in summer. There were clear differences between the building and green landscapes, and the comfort of the building landscape was higher than that of the green landscape.

### The relationship between visual landscape evaluations and subjective environmental evaluations

Some studies have shown that human thermal comfort is related to the environment’s naturality, aesthetic appreciation, and positive experience (Nikolopoulou and Steemers, 2003; Eliasson et al., 2007; Nikolopoulou, 2011). Therefore, it is necessary to evaluate the visual landscape and study its relationship with subjective environmental evaluations. The evaluation results of different visual landscapes are presented in Table 8. In winter, the aesthetics and likability of the different landscapes increased in the following order: water landscape > green landscape > building landscape, and satisfaction and the subjective effect of improving temperature increased in the order of green landscape > water landscape > building landscape. In winter, although the leaves of plants in the green landscape have fallen off and the water area is covered by snow and ice, the subjects still preferred the visual landscapes of the green and water landscapes to the building landscape. Additionally, the green and water landscapes had a clearer effect on subjectively improving the environmental temperature than the building landscape. In the transitional season and summer, the aesthetics, likability, satisfaction, and subjective effect of improving temperature all increased in the following order: green landscape > water landscape > building landscape. In the three seasons, the subjective evaluations of the building landscape were lower than those of the green and water landscapes. Additionally, the subjects believed that green and water landscapes could improve their thermal comfort, and make the effect of temperature more notable. It was also similar to the research results of Ulrich and Smardon, compared with urban landscapes and natural landscapes, that people show a strong tendency toward natural landscapes (Ulrich, 1986; Smardon, 1988).

**TABLE 8:** Visual landscape evaluation results.

Season	Visual landscape evaluation	Building		Green		Water	
		AVG.	S.D.	AVG.	S.D.	AVG.	S.D.
Winter	Aesthetics	0.6	0.72	0.8	0.75	0.9	0.61
	Likability	0.7	0.58	0.9	0.79	1.0	0.67
	Satisfaction	0.8	0.67	1.0	0.69	0.9	0.60
	Subjective effect of improving temperature	–1.2	0.94	–0.1	1.34	–0.2	1.09
Transitional season	Aesthetics	0.7	0.61	0.8	0.81	0.7	0.52
	Likability	0.8	0.61	1.2	0.91	0.9	0.60
	Satisfaction	0.9	0.52	1.1	0.89	0.8	0.53
	Subjective effect of improving temperature	–1.6	0.77	–0.1	1.23	–0.4	0.89
Summer	Aesthetics	0.6	0.60	0.7	0.76	0.7	0.67
	Likability	0.6	0.70	0.9	0.83	0.9	0.67
	Satisfaction	0.6	0.71	0.9	0.80	0.7	0.73
	Subjective effect of improving temperature	–1.4	0.84	0.2	1.27	–0.2	1.05

In order to explore the relationship between visual landscape evaluations and people’s subjective environmental evaluations, the study conducted a correlation analysis of them, as shown in Table 9. There was no significant correlation between the visual landscape evaluation and TSV in winter (*P* > 0.05); satisfaction was significantly positively correlated with TCV (*P* < 0.05); and aesthetics, likability, and satisfaction were all positively correlated with OCV (*P* < 0.05). In the transitional season, there was no clear correlation between the visual landscape evaluation and TSV (*P* > 0.05), while the subjective effect of improving temperature was significantly and negatively correlated with TCV (*P* < 0.01), indicating that the subjects reported lower thermal comfort when the effect of visual landscape on temperature improvement was greater. This may be because the outdoor thermal environment was more comfortable in the transitional season, and people’s sensitivity to other factors affecting comfort will increase, thereby affecting their judgment of TCV (Jin et al., 2019). Additionally, the aesthetics and satisfaction were significantly and positively correlated with OCV (*P* < 0.01). In summer, the aesthetics and likability were negatively correlated with TSV (*P* < 0.05). The higher the aesthetics and likability, the lower the TSV. Additionally, the aesthetics, likability, and satisfaction were all significantly and positively correlated with TCV and OCV (*P* < 0.01). Therefore, the visual landscape evaluations significantly affected the subjective environmental evaluations, and the higher the visual evaluation of the landscape in summer, the better the thermal and overall comfort.

**TABLE 9:** Correlation analysis of visual landscape evaluations and subjective environmental evaluations.

Season	Visual landscape evaluation	TSV		TCV		OCV	
		Correlation coefficient	Significance	Correlation coefficient	Signific-ance	Correlation coefficient	Significance
Winter	Aesthetics	–0.004	0.957	0.021	0.779	0.079	0.286
	Likability	0.107	0.148	0.100	0.178	0.168*	**0.023**
	Satisfaction	0.135	0.069	0.148*	**0.045**	0.239**	**0.001**
	Subjective effect of improving temperature	–0.048	0.518	–0.075	0.315	–0.111	0.136
Transitional season	Aesthetics	0.035	0.632	0.114	0.116	0.228**	**0.001**
	Likability	0.023	0.750	0.075	0.299	0.136	0.059
	Satisfaction	0.041	0.567	0.097	0.181	0.198**	**0.006**
	Subjective effect of improving temperature	–0.040	0.584	–0.222**	**0.002**	–0.089	0.218
Summer	Aesthetics	–0.122*	**0.043**	0.242**	**0.000**	0.246**	**0.000**
	Likability	–0.125*	**0.037**	0.240**	**0.000**	0.270**	**0.000**
	Satisfaction	–0.050	0.404	0.191**	**0.001**	0.208**	**0.002**
	Subjective effect of improving temperature	0.052	0.386	0.066	0.272	0.085	0.210

*Indicates *P* < 0.05, **indicates *P* < 0.01.

Bold font indicates significant analysis results.

## Discussion

### Individual differences in the effects of visual landscape on subjective environmental evaluations

Some existing studies focused on differences in individual characteristics, such as gender, age, clothing, and activity status, and believed that the characteristics affect the response to outdoor physical environments (Lan et al., 2008; Andrade et al., 2011; Chen and Ng, 2012; Yin et al., 2012). Some scholars have shown that individual differences in subjects, such as gender and age, affect thermal comfort evaluation (Jiang et al., 2006; Jin et al., 2019; Xu et al., 2021). So, it is also necessary to discuss the individual differences in the effects of visual landscape on subjective environmental evaluations.

**FIGURE 6:**
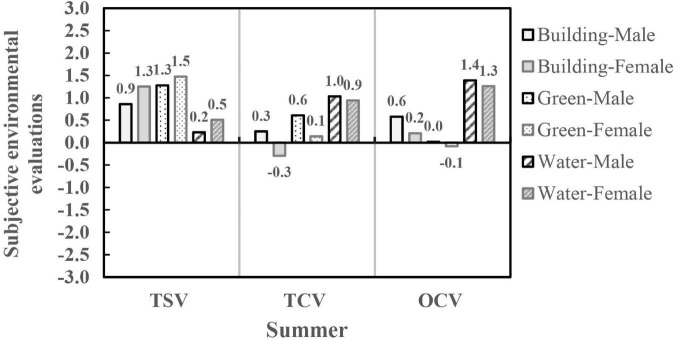
Subjective environmental evaluations of different genders in visual landscape scenes.

The results showed that the effects of visual landscape on the subjective environmental evaluation of different genders were different in summer, including TSV, TCV, and OCV (*P* < 0.05). As shown in Figure 6, the TSV of women in the three visual landscape scenes was slightly higher than that of men, with a difference of 0.2∼0.3. The TCV and OCV of women in the building and greening scenes were significantly lower than those of men, and the differences were about 0.1∼0.5. However, there were no significant differences in TCV and OCV of different genders in the water landscape (*P* > 0.05). It can be found that when the visual landscape was the same, women usually felt relatively hotter and more uncomfortable in summer, indicating that women were more sensitive to the thermal environment in summer. The variation trends in subjective environmental evaluations were relatively consistent in different visual landscape scenes in the same season, and both genders thought that the water landscape made them feel cooler and more comfortable in summer.

**FIGURE 7:**
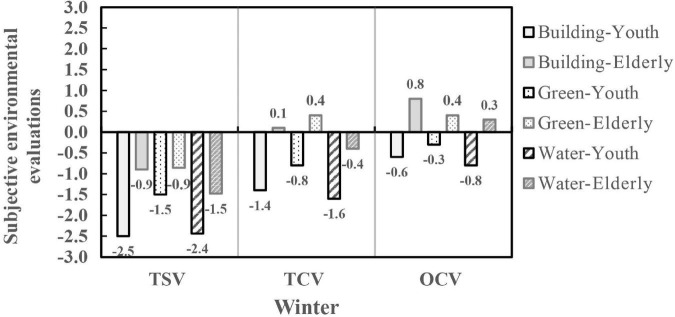
Subjective environmental evaluations of different age groups in visual landscape scenes.

Subjects were divided into youth, middle-aged, and elderly according to age. The results showed that the effects of visual landscape on the subjective environmental evaluation of different age groups were different in winter, including TSV, TCV, and OCV (*P* < 0.01). As shown in Figure 7, The TSV values of the elderly in the building and water landscapes were significantly higher than that of the young, and the differences were about 0.9∼1.5 (*P* < 0.05), but there were no differences in the TSV of the green landscape (*P* > 0.05), indicating that the effects of green landscape on the TSV of different age groups were relatively consistent. The TCV and OCV of the elderly in different visual landscape scenes were significantly higher than that of the youth, with the maximum difference reaching 1.5. This phenomenon might be caused by the long-term living of the elderly in the climatic condition of severe cold regions, and hence the thermal experience and thermal adaptation led to a higher comfort evaluation. In addition, in different visual landscape scenes in the same season, the variation trends in TSV and TCV of different age groups were relatively consistent, and they all felt that the water landscape made them feel colder and more uncomfortable in winter.

### Differences in the effects of visual landscape types on subjective environmental evaluations

In previous studies, some researchers proposed outdoor landscape attributes, such as the ground material, shape, and naturalness, as indirect psychological factors affecting the thermal comfort of people (Knez et al., 2009; Lenzholzer and van der Wulp, 2010). Others explored specific outdoor landscape settings, such as grass, permeable pavements, and street greening, and found that more natural visual landscapes were subjectively more comfortable, which is consistent with the results of this study (Klemm et al., 2015a,b; Mazzotta and Mutani, 2015; Rosso et al., 2015; Schnell et al., 2016). However, previous studies did not explore the effects of different landscape types on subjective environmental evaluations from the perspective of the whole landscape in different seasons. Therefore, three typical visual landscape subjects, i.e., building, green, and water landscapes, were selected to explore the differences in their effects on people’s subjective environmental evaluations in different seasons. However, additional visual landscape components should be selected to elucidate the mechanism by which the visual landscape affects subjective evaluation. This study focuses on the three visual landscape types and comprehensively compares them under different seasonal conditions. The more detailed landscape components of various visual landscapes are not discussed.

### Design measures to improve comfort in the open spaces

According to the results of this study, when improving the environmental quality of urban open spaces, the effects of different visual landscapes on subjective environmental evaluations, especially thermal comfort, should be considered. To an appropriate extent, adding water landscape to the environment can significantly reduce people’s thermal sensation under the same thermal stress level in hot summer and effectively improve thermal comfort and overall comfort. However, the water landscape will also have the opposite effect in the cold winter, reducing people’s thermal sensation and thereby reducing thermal comfort and overall comfort. Therefore, water landscape should be combined with other types of landscape or be seasonally variable to minimize the negative effects in winter. In addition, people’s evaluation of the visual landscape will also affect thermal comfort and overall comfort. Beautiful visual landscapes will improve thermal comfort and overall comfort, so enhancing the aesthetics of urban public open spaces and creating more satisfying and favorite visual landscapes is necessary.

### Limitations and further study

As the experimental investigation was mainly conducted in the outdoor environment in this study, although the experimental conditions were controlled, some factors affected the environmental evaluations and led to a certain extent to standard deviations among the sample data. In order to control variables more effectively, the environmental simulation cabin will be used to simulate the outdoor environment to provide a more stable environment. The study indicates that there were almost no significant differences in the effects of building and green landscapes on subjective environmental evaluations. However, people generally believed that green landscapes significantly affected their environmental perceptions in the survey. Therefore, in-depth and targeted experiments will be conducted on the effects of green landscape on environmental perceptions. In addition, due to the measurement site and time limitations, the average age of the subjects in this study is over 50 years old, and the research results are inclined toward middle-aged and elderly people. Therefore, the sample size will be expanded to cover more subjects of different age groups in the future study.

## Conclusion

Through questionnaire surveys and thermal environment field monitoring in different seasons, an in-depth survey of the effects of different visual landscapes on subjective environmental evaluations and the mechanism of these effects was conducted. The correlation between visual landscape evaluations and subjective environmental evaluations was explored.

(1) The visual landscape only significantly affected TSV in winter and summer. There was no difference between the building and green landscapes; however, both of these landscapes significantly differed from the water landscape. Under the same thermal stress levels, subjects perceived that the water landscape was coldest, followed by the building landscape, and the green landscape was the warmest in winter and summer. From the perspective of thermal experience, the TSV in the water landscape reduced the subjects’ comfort in winter. In contrast, a lower TSV in the water landscape increased the subjects’ comfort in summer.

(2) The visual landscape only significantly affected TCV in winter and summer, while there were no significant differences between the three visual landscapes in the transitional season when the thermal comfort levels were very similar. The thermal comfort in the water landscape was lower than in the building and green landscapes in winter, which were more uncomfortable. In contrast, the TCV of the water landscape was better than those of the building and green landscapes, which were more comfortable in summer. There were no significant differences in TCV between the building and green landscapes in winter and summer.

(3) The visual landscape also significantly affected OCV in winter and summer, and the effects of the visual landscape on OCV were the same as that exhibited on TCV. The OCV in the water landscape was lower than in the building and green landscapes and was more uncomfortable in winter. In summer, the OCV in the water landscape was better than in the building and green landscapes and was more comfortable. There were no apparent differences between the three landscapes in the transitional season, and the OCV values were very similar.

(4) In the three seasons, the visual landscape evaluations of the green and water landscapes were better than in the building landscape. People subjectively believed that the green and water landscapes would improve their thermal comfort and increase the effect of temperature. The visual landscape evaluations significantly affected subjective environmental evaluations in summer than in winter and transitional seasons. The higher the visual evaluation of the landscape, the better the thermal and overall comfort.

## Data availability statement

The raw data supporting the conclusions of this article will be made available by the authors, without undue reservation.

## Ethics statement

The studies involving human participants were reviewed and approved by Academic Ethics Review Committee of Suzhou University of Science and Technology and Academic Ethics Review Committee of Harbin Institute of Technology. The patients/participants provided their written informed consent to participate in this study. The individuals provided their written informed consent for the publication of any identifiable images or data presented in this article.

## Author contributions

JC was responsible for performing experiments and collecting data. YJ was responsible for designing experiments and analyzing data. JC and YJ were responsible for writing the manuscript. HJ was responsible for formulating research plans. All authors contributed to the article and approved the submitted version.

## Abbreviations

TSV: thermal sensation vote
TCV: thermal comfort vote
OCV: overall comfort vote
UTCI: universal thermal climate index.
